# Heterogeneity in the Energy Cost of Posture Maintenance during Standing Relative to Sitting: Phenotyping According to Magnitude and Time-Course

**DOI:** 10.1371/journal.pone.0065827

**Published:** 2013-05-31

**Authors:** Jennifer L. Miles-Chan, Delphine Sarafian, Jean-Pierre Montani, Yves Schutz, Abdul Dulloo

**Affiliations:** Department of Medicine/Physiology, University of Fribourg, Fribourg, Switzerland; University of Santiago de Compostela School of Medicine – CIMUS, Spain

## Abstract

**Background:**

Reducing sitting-time may decrease risk of disease and increase life-span. In the search for approaches to reduce sitting-time, research often compares sitting to standing and ambulation, but the energetic cost of standing *alone* versus sitting is equivocal, with large variation in reported mean values (0% to >20% increase in energy expenditure (EE) during standing).

**Objective:**

To determine the magnitude and time-course of changes in EE and respiratory quotient (RQ) during steady-state standing *versus* sitting.

**Design:**

Min-by-min monitoring using a posture-adapted ventilated-hood indirect calorimetry system was conducted in 22 young adults with normal BMI during 10 min of steady-state standing *versus* sitting comfortably.

**Results:**

This study reveals three distinct phenotypes based on the magnitude and time-course of the EE response to steady-state standing. One-third of participants (8/22) showed little or no change in EE during standing relative to sitting (ΔEE <5%; below first quartile). Of the 14 responders (ΔEE 7–21%), 4 showed sustained, elevated EE during standing, while 10 decreased their EE to baseline sitting values during the second half of the standing period. These EE phenotypes were systematically mirrored by alterations in RQ (a proxy of substrate oxidation), with ΔEE inversely correlated with ΔRQ (r = 0.6–0.8, p<0.01).

**Conclusion:**

This study reveals different phenotypes pertaining to both energy cost and fuel utilization during standing, raising questions regarding standing as a strategy to increase EE and thermogenesis for weight control, and opening new avenues of research towards understanding the metabolic and psychomotor basis of variability in the energetics of standing and posture maintenance.

## Introduction

The modern sedentary lifestyle has long been blamed as a major contributor to the present obesity scourge. Whilst causality remains to be established, excessive sitting has been linked to cardiovascular disease, type 2 diabetes, and all-cause mortality [1]. Furthermore, recent studies have suggested that reducing sitting time to less than 3 h per day may increase life expectancy by 2 years [2], [3]. With the prospect of such positive gains in lifespan, there is now considerable interest in the investigation of simple, effective methods to reduce sitting time. This notion is supported by the observation that obese individuals stand for, on average, 2.5 h less per day than their lean counterparts [4]. Moreover, it has been postulated that, should these obese individuals match the posture allocation of the lean, they could expend an additional 350 kcal/day [4], sufficient to noticeably impact weight loss/gain over time.

In this context, mainstream media is now awash with reports that simply standing burns considerably more calories than sitting, and so-called “standing desks” and “active workstations” are becoming *de rigueur* for socially-conscious employers. Nevertheless, supporting research often compares sitting to a combination of standing and ambulation or fidgeting. Indeed, little research has been undertaken to elucidate the energetic cost of standing alone versus sitting, and that which does is equivocal at best, with large variation in reported mean values. For example, Lawrence and colleagues [5] presented data from a cohort of African women showing no overall increase in energy expenditure (EE) during standing versus sitting (1.26 and 1.25 kcal/min, respectively), whilst Levine *et al* [6] showed an increase of approximately 9% when measured over 20 min (“sitting motionless”  =  5.6; “standing motionless”  =  6.1 kJ/min). A closer inspection of data from the latter study [6], however, reveals a large inter-individual variability, with differences in EE between standing and sitting varying from 0% to 25%. Such heterogeneity in EE during standing compared to sitting was also observed, although not discussed, in earlier studies of Passmore *et al* [7] (range 0% to 28.9%) and Edholm *et al* [8] (range 3.8% to 36%).

This paucity of comparable data is perhaps representative of inconsistency in methodology. The length of standing time over which these measurements are made is often relatively excessive and far from a free-living situation. For example, measurements obtained from a subject standing as motionless as possible for a period as long as 20 min, presented as an integrated mean of the entire standing period may be confounded by stress, or conceal individual variation or variation over time. Furthermore, due to the inherent difficulties involved with measuring EE at different postures, data collected thus far commonly uses mouthpiece and nose-clip, or facemask systems [9], [10] which may add to the stress felt by some subjects and further contributes to the overestimation of the real energy cost of standing.

As such, the present study aimed to determine the extent to which EE, and also substrate oxidation, in a standing position differs from sitting comfortably, by min-by-min monitoring using a ventilated hood indirect calorimetry system, and to determine if/how the energetic cost of standing alters over the course of the 10 min steady-state (SS) standing period.

## Methods

### Subjects

22 young, healthy adult subjects (10 men, 12 women) of European descent participated in the present study, with a mean (±SEM) age of 24±1 y, weight of 67±3 kg, and body mass index (BMI; in kg/m^2^) of 22±1. All subjects were weight-stable, with less than 3% body weight variation in the six months preceding the study. Smokers, pregnant or breast-feeding women, claustrophobic individuals, individuals taking medication, and those with any metabolic disease were excluded. Women were only tested during the follicular phase of their menstrual cycle. The study complied with the Declaration of Helsinki and was approved by the ethical review board of the University of Fribourg; all participants gave written consent.

### Experimental design

**Figure 1:**
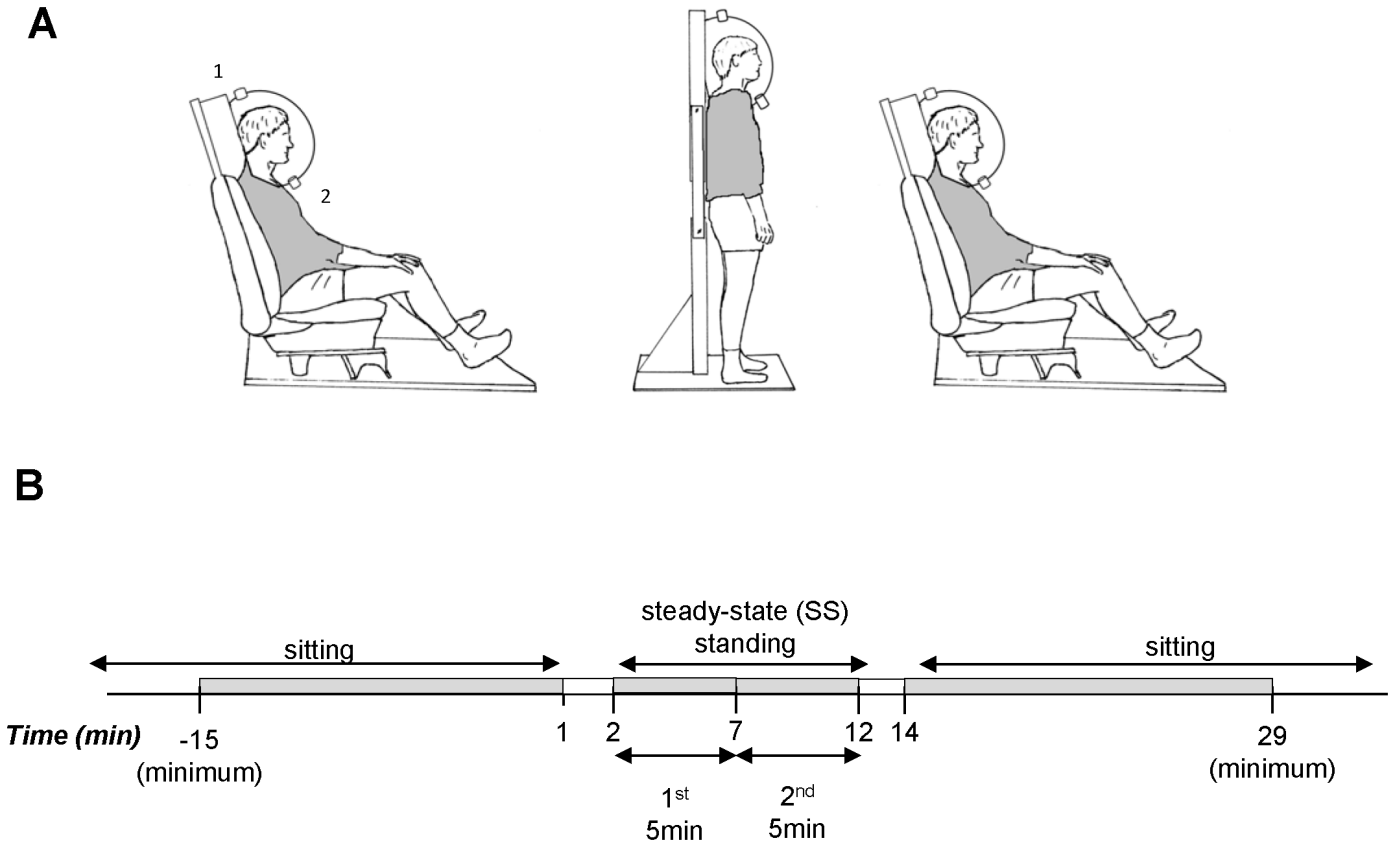
Experimental design and time-line. Schema of experimental design. Posture-adapted ventilated hood indirect calorimetry set-up for sitting and standing measurements (Panel A). The shaded area shows that the area of the subject covered by the veil of the ventilated hood. 1 = air inlet; 2 = air outlet to Deltatrac. Diagrammatic representation of experimental time-line (Panel B). The shaded areas represent the time periods during which minute-by-minute EE and RQ measurements were recorded. A minimum of 15 min of stable measurements were recorded during each sitting period. During postural transition (from sitting-standing, and standing-sitting) the ventilated hood was removed and no measurements recorded. The 10 min steady-state standing period was further divided into two 5 min blocks for data analysis, referred to as “1^st^ 5 min” (minutes 3 to 7, inclusive) and “2^nd^ 5 min” (minutes 8 to 12, inclusive) of the SS-standing period, respectively.

Prior to the day of testing, participants visited the laboratory in order to complete a questionnaire regarding their lifestyle and medical history, and to familiarize themselves with the experimental procedure and equipment. All participants were requested to avoid physical activity, caffeine, and dietary supplements in the 24h prior to testing. On the day of testing, participants arrived at the laboratory at 8h00 following a 12h overnight fast. After the participant voided their bladder, body weight and height were measured using a mechanical column scale with integrated stadiometer (Seca model 709, Hamburg, Germany), and body composition determined by multi-frequency bioelectrical impedance analysis (Inbody 720, Biospace Co., Ltd, Seoul, Korea). EE and respiratory quotient (RQ) were measured using the Deltatrac II ventilated hood system (Datex-Ohmeda, Instrumentarium Corp, Helsinki, Finland) adapted for measurement in a variety of postures; the experimental design is illustrated in **Figure 1**. Participants were seated comfortably in a car seat adapted for calorimetric monitoring, with metabolic measurement conducted until stabilization of EE for at least 15 min, after half an hour of rest. During this period, the participant was instructed to relax and avoid movements. The ventilated hood was then removed and the participant asked to stand relaxed and avoiding movements in front of a wooden frame (supported by a metal base) to which the hood was fixed in a vertical position. This postural transition took a maximum of 2 min, with the measurements of EE and RQ during the transition being excluded from the analysis. A preliminary study showed this to be the length of time required to remove the hood, the subject to change posture, the hood to be replaced, the measurements of EE to equilibrate and the heart rate to stabilize. After transition, EE and RQ were recorded for 10 min, referred to here as the steady-state (SS) standing period. Following a second transition period, measurements were continued during a further sitting period lasting at least 15 min. In order to reduce boredom and accompanying stress, and prevent sleeping, participants were permitted to watch a calm movie or a documentary throughout the metabolic measurements. In addition, heart rate and breathing rate were measured by a wireless physiological monitoring system (Equivital EQ-01, Hidalgo, Cambridgeshire, UK) as indicators of stress.

### Data and statistical analysis

All data are presented as Mean ± SEM unless otherwise stated. The statistical treatment of data, by repeated-measures ANOVA followed by Dunnett’s multiple comparison tests or by linear regressions, were performed using the computer software STATISTIX 8 (Analytical Software, St. Paul, Minnesota, USA).

## Results

### Energy expenditure

**Figure 2:**
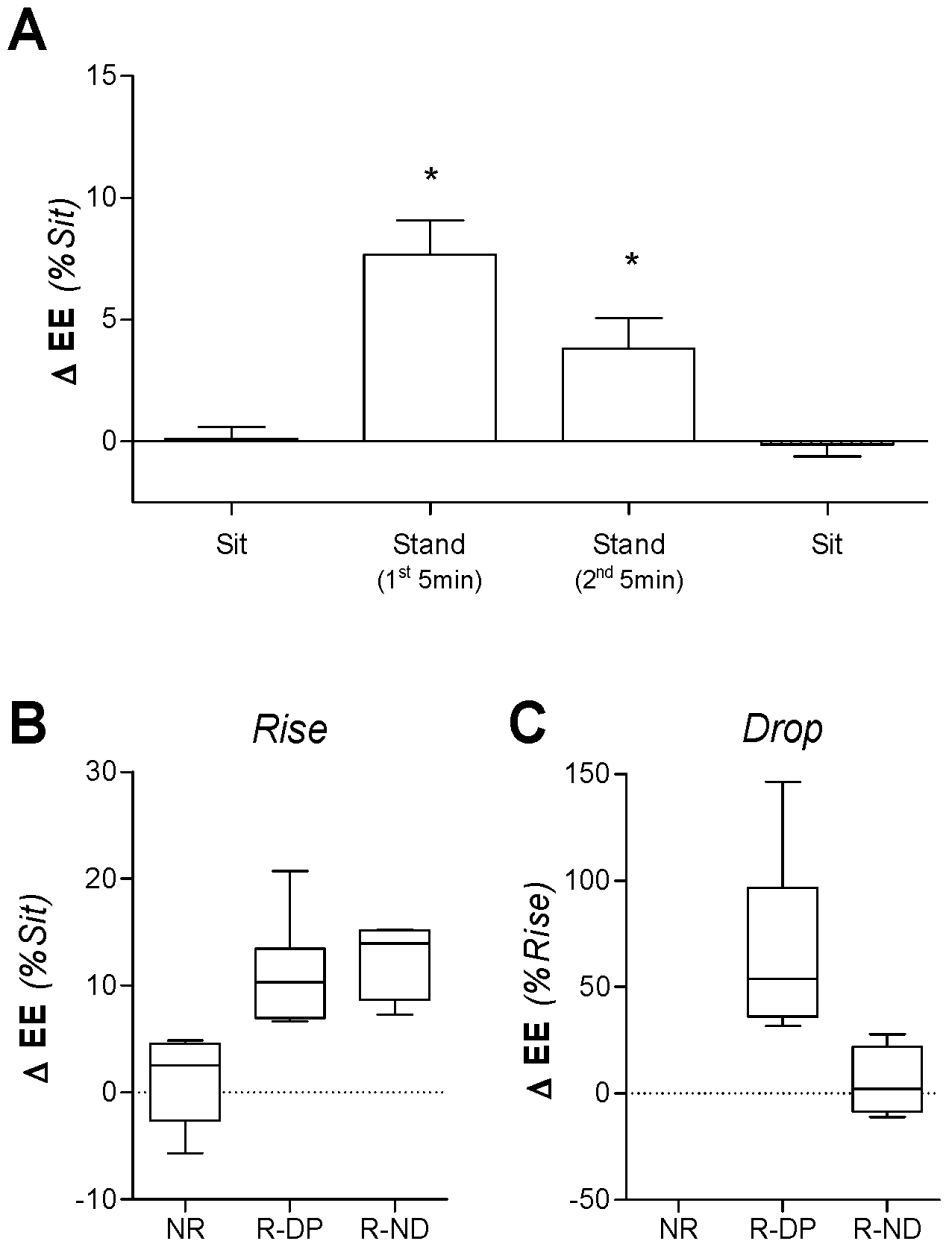
Energy expenditure (EE) during sitting and steady-state (SS) standing. Mean ± SEM energy expenditure (EE) during sitting and steady-state (SS) standing, expressed as percentage change relative to mean sitting EE (Panel A); *statistically significant from baseline as assessed by repeated-measures ANOVA followed by Dunnett’s multiple comparison tests. In the present study the percentage change from the mean sitting value to the mean of the first 5 min of the SS-standing period is referred to as “rise” from sitting value. The percentage change from the mean of the first 5 min to the mean of the second 5 min of the SS-standing period is referred to as “drop” to sitting value. Box and whisker plot comparing rise (Panel B) and drop (Panel C) for each EE response group. NR = Non-Responders, R-DP = Responder Droppers, R-ND = Responder Non-Droppers.

Overall, there was a significant increase in mean EE over the entire 10 min SS-standing period compared to mean sitting EE (4.28±0.18 *vs* 4.05±0.16 kJ/min, p<0.001). However, in order to take into account individual differences in EE, these data were further analyzed in terms of percentage change from sitting, with an average increase in EE of 5.7±1.2% during the entire 10 min SS-standing period (range –0.9% to +15.4%). This analysis also showed that EE (as a percentage of sitting EE) was significantly higher during the first 5 min of the SS-standing period compared to the second 5 min (7.7±1.4 *vs* 3.8±1.3%, p<0.01; **Figure 2A**).

While absolute sitting EE values were lower in females when compared to their male counterparts (Male: 4.70±0.1; Female: 3.50±0.13 kJ/min; p<0.001), overall there was no significant difference between these two gender groups in terms of percentage change (p = 0.86).

However, across both genders, we observed an apparent heterogeneity in the EE response to steady-state standing relative to sitting, and furthermore, in the time-course of this response across the 10 min SS-standing period. Therefore, participants were classified into three groups according to the following criteria (**Figures 2B and 2C** **)**:

1. Non-Responder (*n* = 8): Those who showed little or no change in EE (rise < first quartile, which is equivalent to a rise in EE of <5%) during SS-standing period relative to sitting.
2. Responder Dropper (*n* = 10): Responders who increased EE (rise > first quartile, which is equivalent to a rise in EE of >5%) during first 5 min SS-standing period relative to sitting but subsequently decreased EE in the second 5 min of this standing period (drop >30% of the rise in EE during first 5 min SS-standing period).
3. Responder Non-Dropper (*n* = 4): Responders who increased EE (rise > first quartile, which is equivalent to a rise in EE of >5%) during first 5 min SS-standing period relative to sitting and who maintained an elevated EE throughout the entire SS-standing period (drop <30% of the rise in EE during first 5 min SS-standing period).

**Figure 3:**
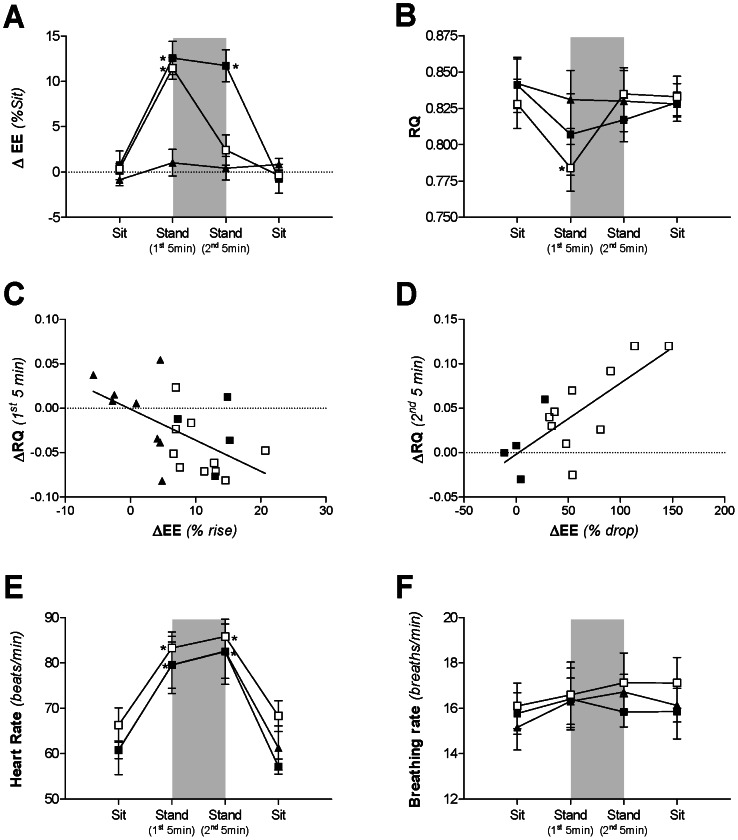
Comparison of energy expenditure, heart rate and breathing rate. Comparison of mean ± SEM energy expenditure (EE, Panel A), RQ (Panel B), heart rate (beats/min, Panel E), and breathing rate (breaths/min, Panel F) for each EE response group: Non-Responders (▴), Responder Droppers (□), Responder Non-Droppers (▪). The shaded area indicates the steady-state standing period. *statistically significant from baseline as assessed by repeated-measures ANOVA followed by Dunnett’s multiple comparison tests. Panels C and D indicate the relationships between the change in RQ *vs* change in EE during the first 5 min (percentage “rise” from sitting value, Panel C), as well as *vs* change in EE during the second 5 min (percentage “drop” to sitting value, Panel D) of the 10 min steady-state standing period. ΔEE (% rise) *vs* ΔRQ (1^st^ 5 min): r = 0.56, p<0.02; ΔEE (% drop) *vs* ΔRQ (2^nd^ 5 min): r = 0.77, p = 0.001. Non-Responders (▴), Responder Droppers (□), Responder Non-Droppers (▪).

These three groups are compared in **Figure 3A**. By definition, those classified Non-Responders had a significantly lower mean rise in EE than the responders (1.0±1.5% *vs* 11.5±1.1%, p<0.001), and the Responder Non-Dropper group a significantly lower drop in EE than the Responder Dropper group (–5.2±8.2% *vs* –69.0±12.1%, p<0.001). During the second 5 min SS-standing period, the mean EE of the Responder Non-Dropper group remained significantly higher than that measured during sitting (p = 0.01), while the mean EE of the Responder Dropper group had returned to baseline sitting values (NS).

No significant correlation was found between the change in EE (as %rise or as %drop) and anthropometry (body weight, height, sitting height), BMI or body composition (body fat, fat-free mass (FFM), skeletal muscle mass, ratio of fat-to-FFM).

### Respiratory Quotient

Overall, when all subjects were pooled, RQ was significantly lower when integrated over the entire 10 min SS-standing compared to the sitting period (0.817±0.010 *vs* 0.833±0.008, p<0.01), with no difference between the first 5 min and second 5 min of this standing period (0.805±0.012 *vs* 0.830±0.011, p = 0.07).

A comparison of RQ across the three groups, classified by EE response as described above, is shown in **Figure 3B**. The Non-Responder group showed no significant change in RQ during SS-standing, whereas responders showed a significant decrease in RQ during the first 5 min of the standing period compared to sitting (p<0.01). During the second 5 min of the 10 min SS-standing period, the RQ of all groups was not significantly different from sitting values; nonetheless RQ in the Responder Non-Dropper group tended to be lower than in the other two groups.

Further analysis of RQ response during the first 5 min of SS-standing showed an inverse correlation between %rise in EE and change (decrease) in RQ (r = 0.56, p<0.005; **Figure 3C**), and also positive correlation between %drop in EE and change (increase) in RQ during the second 5 min of the SS-standing period (r = 0.77, p = 0.001; **Figure 3D**).

### Heart rate and breathing rate

Standing significantly increased heart rate compared to sitting (82.6±2.9 *vs* 63.7±2.5 beats/min, p<0.001). This change was consistent across all three EE groups, including the Non-Responders (**Figure 3E**). Conversely, breathing rate did not significantly change with posture in any group (mean sit: 16.1±0.6; mean stand: 16.6±0.7 breaths/min; **Figure 3F**).

## Discussion

The present study supports the argument that, in general, standing elevates EE above sitting values. However, unlike previous studies which have presented standing EE as an integrated mean, this study using min-by-min monitoring via a ventilated hood indirect calorimetry system to determine the time-course of EE change across the entire SS-standing period also reveals large heterogeneity across the study population in the amplitude and time course of EE response to SS-standing compared to sitting, with three phenotypes identified.

Of the 22 participants, approximately one third of the participants (*n* = 8) showed little or no change in EE during SS-standing relative to sitting (Non-Responder group, EE rise <5%). Also surprisingly, of the responders, 10 of 14 participants decreased their EE to baseline sitting values during the second 5 min of the 10 min SS-standing period (Responder Dropper group). Furthermore, these changes in EE were systematically mirrored by alterations in RQ, with greater increases in EE during the first 5 min of the SS-standing period correlated with greater decreases in RQ (suggesting a shift in favor of fat oxidation), and with a greater drop in EE towards baseline sitting values correlated with greater recovery in RQ in the second 5 min of the SS-standing period.

It is unlikely that these differences were due to an elevated or sustained stress/anxiety response in the Responder Non-Dropper group, or conversely a decreased stress/anxiety response in the Non-Responder group, as the length of the standing period was well tolerated by all subjects, and shorter in duration than that used in comparable studies. Participants were instructed to stand “naturally” and so were able to shift weight between legs as necessary, and the ventilated canopy system allowed the subject to feel as comfortable as possible without any of the inherent difficulties associated with facemask or mouthpiece and nose-clip systems. Furthermore, in order to identify possible stress/anxiety, heart rate and breathing rate were measured continuously throughout the experiment. Breathing rate showed no change across the protocol, clearly indicating that the decreased RQ could not be attributed to lower ventilatory response (hypoventilation), and the increased heart rate during SS-standing was identical across all groups. Furthermore, no correlation was found between EE response and anthropometry (body weight or height), BMI or body composition (body fat, FFM, skeletal muscle mass, or Fat:FFM).

This observation that individuals may show markedly different EE responses to the same challenge is supported by a plethora of research investigating the energetic cost of standardized activities amongst developing and subsistence-level populations [11], [12], and across ethnic groups [13], [14]. However, all the participants in the present study were young, healthy Caucasian Europeans, and therefore we postulate that the differences more likely lie within the musculoskeletal efficiency in postural maintenance. Given that the observed changes in EE during SS-standing compared to sitting were mirrored so clearly by changes in RQ, it would appear that postural muscles (which are largely slow oxidative in metabolic profile) may be differentially activated across the three phenotypic groups identified here. Therefore further studies involving electromyographic measurements and the comparison of blood markers of substrate turnover would be of particular interest.

Whilst the findings of this study are intriguing, it was not without its limitations. Due to the observation of different phenotypes of EE during SS-standing, and hence the subdivision of the study population, larger-scale studies are necessary. In addition, we chose to use a ventilated hood system to minimize discomfort for the participants, however this method did not allow for measurement during the 2 min period of postural transition (from sitting to standing and *vice versa*). During postural change a number of complex physiological processes are undertaken to regulate the body’s cardiovascular and musculoskeletal response. Individual differences in the mechanisms or magnitudes of these responses may lead to the phenotypic patterns of EE change we observed in this study and as such this transition period warrants further, in-depth investigation.

Standing is one of the simplest conscious physical activities in which we partake, but our metabolic response appears to be anything but simple. Whilst it seems some of us may benefit from a sustained, 10% increase in EE, others show only an acute increase, or little or no increase in EE at all. Even in those showing the highest rise in EE during standing (i.e. the Responder Non-Droppers), increasing time spent standing for postural maintenance *per se* by 2.5 h per day would amount to an excess energetic cost of <20 kcal/day above sitting, far less than the 350 kcal/day claimed by others [4], which most likely represents fidgeting rather than the effect of standing posture *per se*. Alternatively, some individuals might spontaneously need to fidget more than others in order to maintain posture while standing. Therefore, with such immense interest in methods to increase our daily physical activity in the modern sedentary environment [15], our results raise questions regarding standing as a strategy to increase EE for weight control in many individuals and open new avenues for research into better understanding the metabolic and psychomotor basis of variability in the energetics of standing and posture maintenance.
